# PCD Advances Recommendations From First-Ever External Review

**DOI:** 10.5888/pcd15.180414

**Published:** 2018-09-06

**Authors:** Leonard Jack

**Figure:**
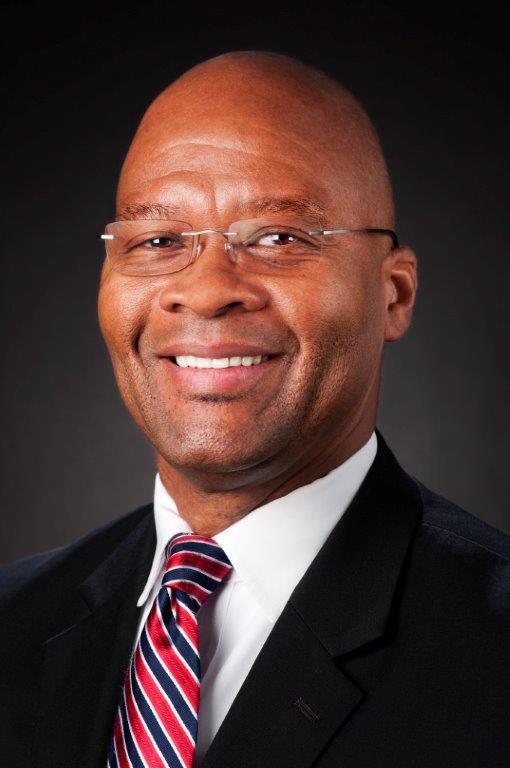
Leonard Jack Jr, PhD, MSc

In April of this year, the Editor in Chief column provided PCD readers with information on how the journal used recommendations from its first-ever external review to enhance the journal’s worldwide usefulness to researchers, practitioners, and policy makers. The journal refined its vision and mission statement to reflect its commitment to serving as an influential journal in the dissemination of proven and promising public health findings, innovations, and practices. The review’s panel of experts strongly encouraged the journal to move away from publishing manuscripts that solely described partnerships, collaborations, and coalition building. Instead, the journal was encouraged to focus more on complementing the journal’s rich body of published work on epidemiological studies with attention to evaluating population-based interventions and policies. Since sharing recommendations from the expert panel with our readers, PCD has worked hard to advance those recommendations. This Editor in Chief column provides information on how the journal is increasing the number of publications focusing on population-based interventions, our upcoming 2018 collections, and the future direction of the Student Research Paper Contest.

## Article Types Focusing on Population-Based Interventions

Over the past year, PCD has introduced 2 new article types to increase attention to evaluating population-based interventions and policies. First, PCD introduced the article type Implementation Evaluation, which recognizes that public health and clinical interventions are often collaborative, multifaceted, multicomponent, and multisite with diverse participants and stakeholders. The Implementation Evaluation article type requires that authors provide insight on how the evaluation approach could be improved. This article type also shares with readers ways to facilitate diffusion and uptake of effective interventions in comparable real-world settings. Second, PCD introduced the article type Program Evaluation Brief, which is a condensed version of the Implementation Evaluation article type. These short articles focus on understanding how various aspects of activities related to program implementation, program performance, health policy, or system change can influence health.

Both article types are designed to allow authors an opportunity to share lessons learned about factors or circumstances that contributed to or posed challenges to their research, which will help the field identify the most effective population health approaches. However, they are not designed to focus exclusively on providing program descriptions or theoretical framework descriptions; they also are not literature reviews, opinion pieces, commentaries, or essays.

Since making these 2 options available, as of July 2018, PCD has received over 30 related manuscripts. This early response indicates that these article types resonate with PCD contributors. Expanding PCD’s scope to include more research in these areas will certainly help PCD achieve its goal of publishing quality content focusing on the effectiveness of population-based interventions to improve population health.

## 2018 PCD Collections

Also in April of this year, PCD refined and published on its website 4 content areas of greatest interest to the journal:

- Development, implementation, and evaluation of population-based interventions to prevent chronic diseases and control their effect on quality of life, illness, and death.
- Behavioral, psychological, genetic, environmental, biological, and social factors that influence health.
- Interventions that reduce the disproportionate incidence of chronic diseases among at-risk populations.
- Development, implementation, and evaluation of public health law and health-policy–driven interventions.

To assist the journal in expanding its content in these 4 areas, PCD announced calls for papers for 2 collections:

1. **Population Health, Place, and Space: Spatial Perspectives in Chronic Disease Research and Practice**. Authors are encouraged to submit manuscripts for consideration that focus on various ways in which geographic information systems and spatial analyses are used to enhance chronic disease research and public health practice. PCD is excited about publishing articles that offer insight into the role of place and space in shaping the distribution of chronic disease and that inform public health responses for chronic disease prevention and treatment. Manuscripts for this collection are due August 31, 2018. For more information, please visit https://www.cdc.gov/pcd/announcements.htm#call.
2. **Health Care Systems, Public Health, and Communities: Population Health Improvements.** Manuscripts accepted for this collection will focus on research, evaluation, and other work describing innovative and effective work to link health care and community health in ways that improve population health. Over the past decade, there have been various innovative community-driven and clinically driven prevention strategies (primary and secondary) designed to prevent and reduce the burden of chronic conditions worldwide. PCD invites manuscripts that provide timely information on jointly implemented public health and health care efforts to improve population health. Manuscripts should be submitted to PCD on or before November 16, 2018. For more information, please visit https://www.cdc.gov/pcd/announcements.htm#callformanu.

Manuscripts submitted in response to these 2 calls for papers will be reviewed and, if accepted, published on a rolling basis. Articles will be assembled into PDF collections accessible on the PCD website after all accepted articles for each collection have been published. PCD welcomes submissions that address a broad range of health conditions (eg, hypertension, diabetes, cancer, obesity, asthma, arthritis, oral health, reproductive health, substance abuse, mental health) and their risk factors, with application to a wide range of settings. Manuscripts must follow the instructions for PCD article types (htts://www.cdc.gov/pcd/for_authors/types_of_articles.htm). Further information on submitting a manuscript is available in PCD’s Author’s Corner (https://www.cdc.gov/pcd/for_authors/index.htm).

## Student Research Paper Contest

### 2018 Winners

PCD received a record number of 109 student papers for this year’s Student Research Paper Contest. Four papers were selected as winners in 3 categories: doctoral, graduate, and undergraduate. Two winners were selected in the graduate category. The winning papers and podcast interviews for each winning article will be published in PCD later this year. Please join us in congratulating this year’s student winners:

- **Doctoral Winner: Brittney Keller-Hamilton, College of Public Health, Ohio State University — **Tobacco and Alcohol on Television: A Content Analysis of Male Adolescents’ Favorite Shows
- **Graduate Winner: Calla Holzhauser, Mathematics and Statistics Department, South Dakota State University — **Forecasting Participants in the All Women Count! Mammography Program
- **Graduate Winner: Fei Gao, Department of Public Health, Brody School of Medicine, East Carolina University — **Gestational Diabetes and Health Behaviors Among Women: National Health and Nutrition Examination Survey, 2007–2014
- **Undergraduate Winner: Leigha Vilen, Thurston Arthritis Research Center, University of North Carolina at Chapel Hill — **Education Attainment, Health Status, and Program Outcomes in Latino Adults with Arthritis Participating in a Walking Program

Conducting the student paper contest annually requires a tremendous amount of coordination with review panel chairs and committees, reviewers, and student authors. There are rounds of reviews and decision making to determine which manuscripts advance through each stage of the review process. Not all student papers are selected as winners. However, the contest does allow student authors of nonwinning papers an opportunity to be published once all publication requirements and expectations are met. The quality of submissions was superb this year, and PCD intends to publish 30% of the 109 papers submitted for consideration.

### Future directions for the contest

This was our most successful year so far, and the record number of submissions demanded that PCD expend its resources to the limit. PCD created multiple review committees from our editorial board, solicited additional reviewers, and processed a record number of submissions and revisions through our editorial office. Recognizing that it was likely that submissions to our contest would increase each year, we had concerns that we would not be able to continue providing the same level of attention and mentoring to students, which was the mission of the student contest since its inception. Instead of holding a contest every year, we have opted to hold contests on a less frequent basis that focus on an evolving research area or topic of interest. Moving forward, PCD will conduct it student paper contest every 3 years. The next student paper contest will take place in 2021. By focusing attention on emerging areas of research of importance to students, we can continue to ensure that the contest remains relevant to students and that we can prepare resources in advance to ensure that students get the same quality of attention, review, and mentoring that they have come to expect from PCD.

## Conclusion

PCD has maintained its focus on publishing research that provides insight into the identification and use of innovative and rigorous evaluation approaches to determine the impact of interventions that address chronic disease prevention and control. We hope potential authors will consider submitting manuscripts under our new article types and under our calls for papers. Please visit the journal’s website to learn more about recent publications, upcoming collections, podcasts, and innovations adopted by the journal.

